# An Edible Biohybrid Platform Accomplishes In Situ Fenton‐Mediated Enteral Nanoplastics Aging and Excretion

**DOI:** 10.1002/advs.75918

**Published:** 2026-05-30

**Authors:** Su Zhou, Anran Yan, Haowei Guo, Ping Chen, Qiang Chu

**Affiliations:** ^1^ Tea Research Institute Zhejiang University Hangzhou China

**Keywords:** edible biohybrid platform, fenton reaction, intestinal regulation, nanoplastic, nanotoxicology

## Abstract

Ingested nanoplastics (NPs) readily translocate across the intestinal barrier due to their small size, posing a pervasive threat to human health. Current mitigation approaches, constrained to environmental water purification or postexposure injury alleviation, entail unavoidable NPs ingestion or organism damage. Effective strategies to impede NPs internalization at the lumen stage and promote safe elimination remain limited. Here, an edible biohybrid platform, composed of a natural polyphenol reductant, redox‐active ferric ions, and H_2_O_2_‐generating *Enterococcus faecalis*, is developed to trap NPs within the intestine. The platform enables persistent in situ generation of hydroxyl radicals in the intestine through Fenton reactions, accelerating the oxidative aging and subsequent NP agglomeration into micrometer‐scale clusters that exceed the physiological pore‐size limit of intestinal barrier. In an in vitro intestinal barrier model, the platform achieved a 96.02% NPs clearance efficiency within 24 h. Daily oral co‑administration effectively blocked NP penetration into lamina propria, alleviated intestinal inflammation, tight‑junction disruption, and mucosal damage in mice, as well as a protective effect further corroborated by the diminished NP fluorescence in *C. elegans*. Validated with both polystyrene and polypropylene NPs, this strategy of directed regulation of intestinal NP behaviors offers a green and generalizable approach to combat the NP exposure hazards.

## Graphical abstract

An edible biohybrid platform triggers nanoplastics (NPs) aging and agglomeration by in situ generating hydroxyl radicals within the intestine. The enlarged NPs are unable to penetrate the intestine and are subsequently excreted. Simultaneously, probiotic biofilms reinforce the intestinal barrier. This platform effectively repairs intestinal damage and prevents potential systemic toxicity.

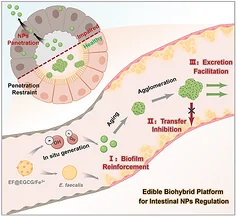

## 1 Introduction

Plastic particles have become an unintended but persistent component of the human exposome, with an estimated daily human intake of 11.5–23.7 mg/kg (safe dose of approximately 0.25 mg/kg) [1], have raised growing toxicological concerns [2, 3, 4, 5]. Nanoplastics (NPs), owing to their small size and high dispersibility, readily translocate across the intestinal epithelium into systemic circulation and accumulate in organs including the liver, kidneys, and brain [6, 7, 8, 9]. The intestine, being the largest immune organ in close contact with the external environment, is both a primary target and a crucial defense line [10]. Existing countermeasures largely focus on removing NPs from water sources or treating organ damage after systemic absorption. Yet NPs are ubiquitous in the environment, and complete removal from drinking water remains unrealistic, while postexposure therapies cannot reverse preexisting injury. A critical but underexplored opportunity, therefore, lies within the gastrointestinal lumen, where ingested NPs remain accessible for interception after ingestion but before epithelial translocation. Strategies that intervene at this preabsorptive stage by altering NP colloidal state and restricting mucosal contact represent a promising direction for NP mitigation, yet such edible and safe platforms remain extremely limited [11, 12, 13].

Notably, the intestinal migration behavior of NPs is size‐dependent [14]. Smaller NPs (∼<100 nm) readily penetrate the mucus layer and cellular barriers, whereas larger NPs (more than several hundred nanometers in dimension) are discharged with the food residues [3, 15, 16, 17]. This suggests that inducing NPs agglomeration to increase size is key to inhibiting their penetration. Research indicates that NPs undergo aging under oxidation, with the surface becoming rugged, and some appearing to flake off. The increased specific surface area and more exposed groups would enhance the absorption capacity towards persistent organic pollutants and heavy metals. Agglomeration subsequently occurs, resulting in larger particles that can be blocked by multi‐barriers, and eventually eliminated from the body [18, 19, 20, 21]. However, NPs aging under natural environmental conditions require decades to centuries to complete [22]. Achieving equivalent oxidation rapidly inside the gut would enable substantially enhanced NPs elimination. Oxidative agents intended for enteral NP aging must therefore combine high reactivity with a short lifespan and must be generated locally in the immediate vicinity of NPs to avoid diffuse tissue injury.

Hydroxyl radicals (•OH) possess an exceptionally high oxidation potential (2.80 V vs. SHE) and reaction rate constants approaching the diffusion‐controlled limit (∼10^9^ to 10^10^ M^−1^s^−1^), driving NP aggregation into the 200–500 nm hydrodynamic range [23]. The Fenton reaction is among the most widely adopted methods for •OH generation, but is strictly dependent on H_2_O_2_, which is scarce and spatially heterogeneous in the intestine [24, 25, 26]. Based on the demand for edibility and safety, the integration of living microorganisms with green functional materials presents an elegant solution to the H_2_O_2_ supply problem [27].

In this study, a natural polyphenol epigallocatechin gallate (EGCG)‐Fe^3+^‐modified *E. faecalis* is used as an edible biohybrid platform (EF@EGCG/Fe^3+^) for hindering NPs translocation across the intestinal barrier and facilitating excretion. *Enterococcus faecalis* (*E. faecalis*, EF), a Gram‐positive facultative anaerobe and probiotic, continuously produces extracellular H_2_O_2_ at up to millimolar local concentrations, while simultaneously colonizing the mucosal surface and forming a reinforcing biofilm [28, 29]. EGCG, a tea catechin abundant in phenolic hydroxyls, chelates two Fe(III) ions via its galloyl and pyrogallol rings (stability constant 43.76) and enables continuous Fe^3+^/Fe^2+^ cycling to sustain Fenton reaction [30]. The polyphenol coating further promotes mucoadhesion through hydrogen bonding and π–π stacking with intestinal mucins while supporting barrier repair [31]. Upon reaching the intestine, the colonized *E. faecalis* enables continuous in situ H_2_O_2_ production that reacts with the EGCG and iron to generate •OH, which introduces carbonyl and carboxyl groups onto NP surfaces, driving inter‐particle conjugation and agglomeration into micrometer‐scale clusters [32]. These clusters exceed the physiological size limit of the intestinal barrier, and the co‐established *E. faecalis* biofilm further restricts residual NP permeation, ultimately enhancing fecal NP excretion and mitigating NP‐induced intestinal toxicity. Departing from pre‐exposure removal from environmental matrices or postdamage pharmacological therapy, this study targets ingested NPs that remain within the gastrointestinal lumen, proposing a directed regulation of intestinal NP transformation and migration behaviors as a green, safe, and generalizable strategy against NP health hazards.

## 2 Results and Discussion

### 2.1 Characteristics of EF@EGCG/Fe^3+^ Hybrids

**FIGURE 1:**
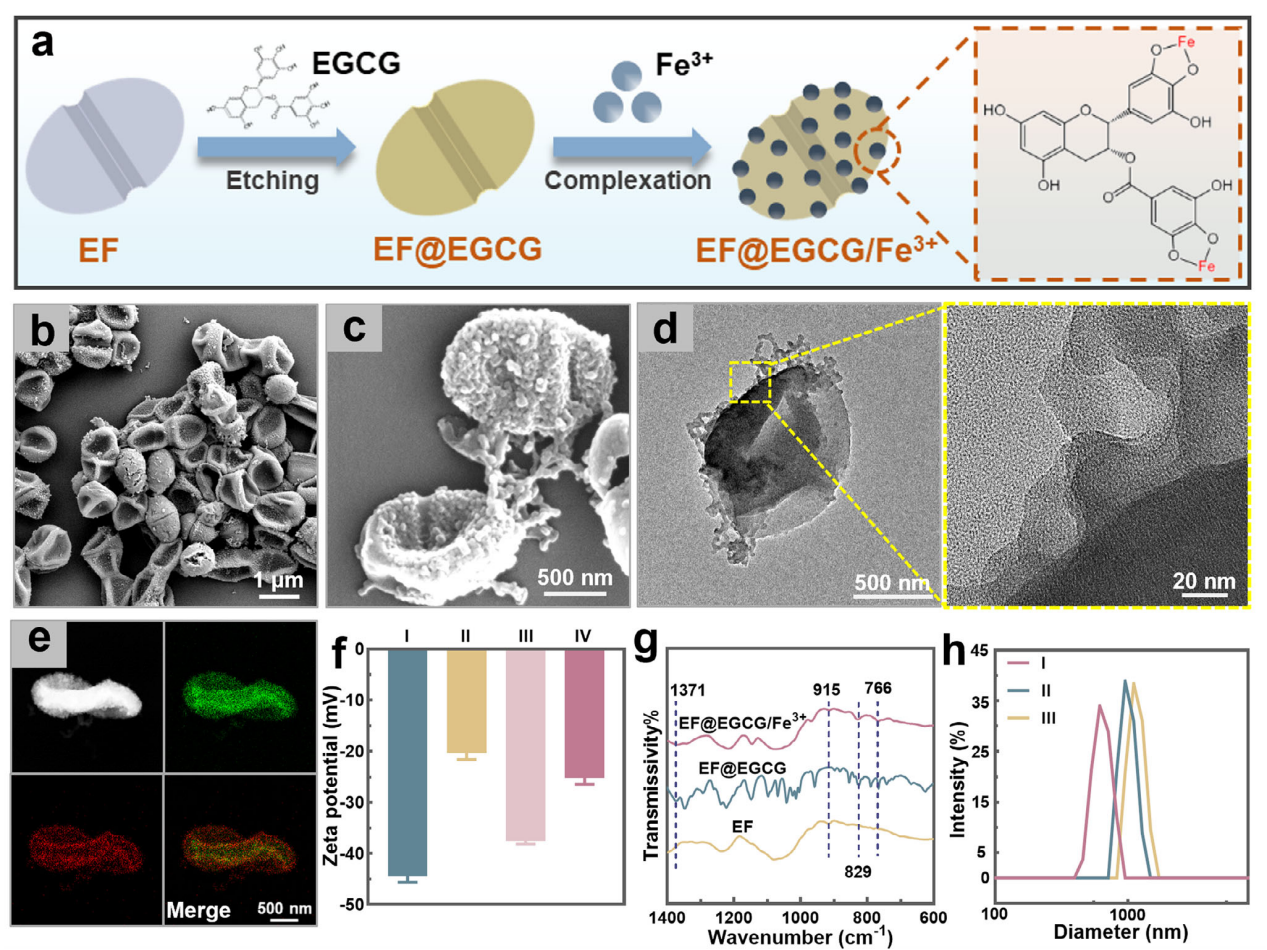
Characteristics of the EF@EGCG/Fe^3+^ biological hybrid platform. (a) Schematic illustration of the synthesis procedure for EF@EGCG/Fe^3+^. (b) SEM image of EF@EGCG/Fe^3+^. (c) Magnifying SEM image of EF@EGCG/Fe^3+^. (d) TEM image of EF@EGCG/Fe^3+^. (e) EDS mapping images of EF@EGCG/Fe^3+^. (f) Zeta potential of I: *E. faecalis*, II: EGCG, III: EF@EGCG, and IV: EF@EGCG/Fe^3+^. (g) FT‐IR spectrum of *E. faecalis*, EF@EGCG, and EF@EGCG/Fe^3+^. (h) Size distributions of I: *E. faecalis*, II: EF@EGCG, and III: EF@EGCG/Fe^3+^.

A metal‐polyphenol framework composed of EGCG and Fe^3+^ was assembled onto the surface of *E. faecalis* through a two‐step etching and complexation procedure, yielding EF@EGCG/Fe^3+^ hybrids. (Figure 1a and Figure S1). SEM images revealed visible shrinkage of *E. faecalis* caused by moisture loss during sample preparation, with spherical mineral particles approximately 50 nm in diameter deposited densely and uniformly on the cytoderm, producing a porous mineral crust (Figure 1b,c). TEM further displayed the close junction between the inorganic particles and the bacterial surface, particularly at high magnification, where fractions with disparate mass‐thickness contrast were observed in intimate contact (Figure 1d and Figure S2a). EDS mapping presented the location of diverse chemical elements, with green oxygen element gathering in the central domain and red iron element mainly situated in the outer layer, forming an enclosed “armor” (Figure 1e). The comparable electronegativities of *E. faecalis* and EGCG (−44.5 mV and −20.33 mV, respectively) illustrated that their interaction was not dependent on electrostatic force (Figure 1f). Research shows that microorganisms can serve as bio‐templates for chemical encapsulation due to numerous functional groups on the surface [33]. For instance, catechol‐containing compounds such as dopamine exhibit natural adhesiveness toward cells through covalent and noncovalent linkages, and the multiple catechol groups of EGCG confer enhanced binding affinity to the cell surface without compromising viability [34, 35, 36]. This suggests a specific interaction mode of EGCG towards bacteria. The subsequent addition of Fe^3+^ induces chelation with EGCG, resulting in the formation of a 20 nm thick porous shell and the neutralization of partial electronegativity. Successful attachment of EGCG and Fe^3+^ to *E. faecalis* is evident from the presence of mutual functional groups observed from the FT‐IR spectrum, such as peaks at 766, 829, 915, and 1371 cm^−1^, and the decreased absorption peaks from EF@EGCG to EF@EGCG/Fe^3+^ reflected the covering of specific functional groups (Figure 1g and Figure S3c). The layer‐by‐layer deposition of the substances was reflected by a stepwise increment in size through DLS analysis (Figure 1h). In addition, the surface armoring also altered the crystal structure and optical properties of *E. faecalis* as depicted in Figures S2b and S3a,b. Consequently, these findings provide compelling evidence for the excellent integration of EGCG‐Fe^3+^ coatings towards living *E. faecalis*, leading to the synthesis of a stable hybridized platform EF@EGCG/Fe^3+^.

### 2.2 Extracellular Properties Characterization of EF@EGCG/Fe^3+^

**FIGURE 2:**
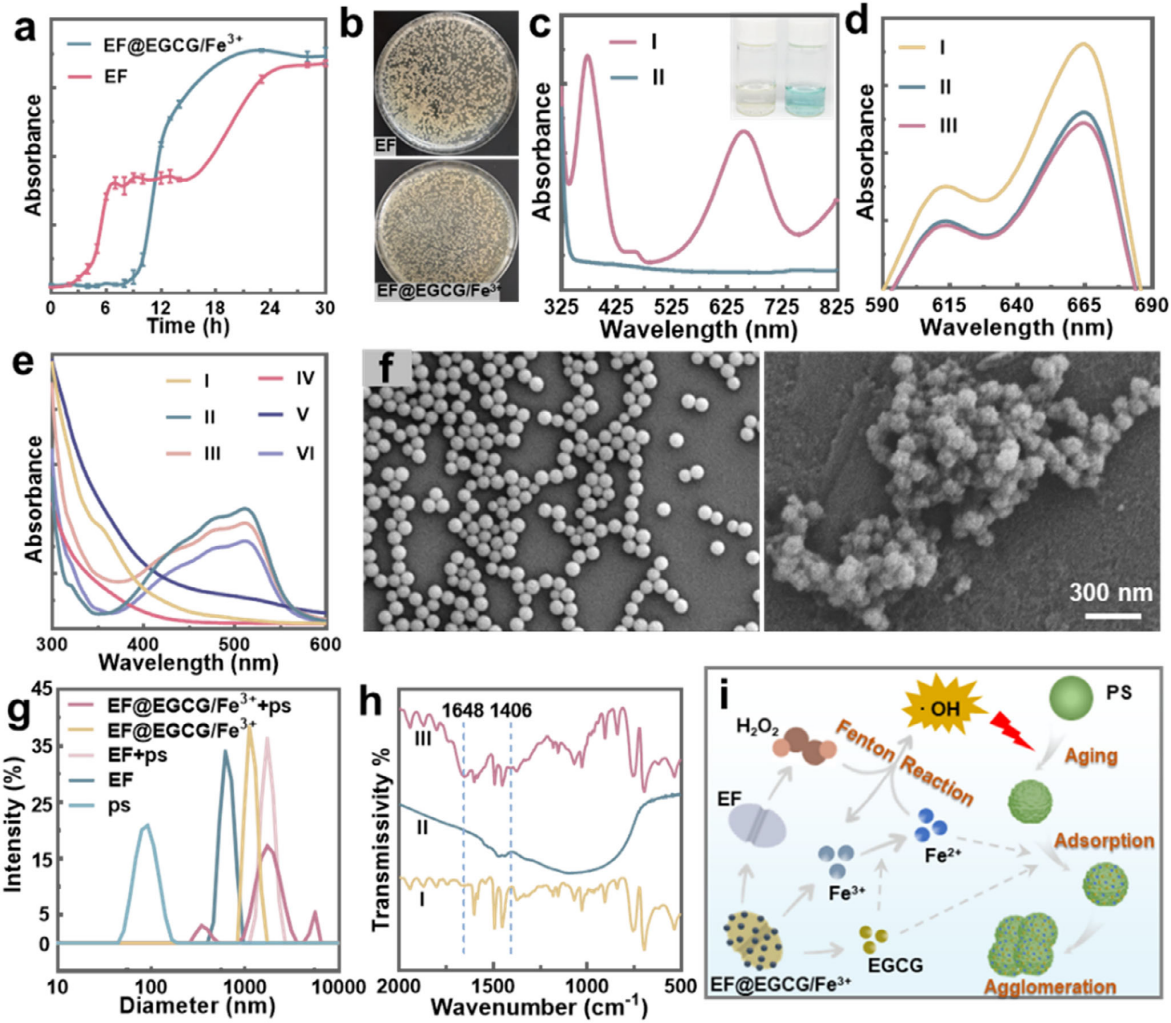
Extracellular chemodynamic performance of EF@EGCG/Fe^3+^. (a) Growth curves of *E. faecalis* and EF@EGCG/Fe^3+^. (b) Bacterial colony images of *E. faecalis* and EF@EGCG/Fe^3+^. (c) UV–vis absorbance spectra and corresponding optical photographs (inset) of TMB aqueous solution with the addition of pure medium (Group I) and *E. faecalis* culture medium (Group II). (d) UV–vis absorbance spectra of TMB solution with I: water, II: 1/4 diluted EF@EGCG/Fe^3+^ culture medium, and III: EF@EGCG/Fe^3+^ culture medium. (e) UV–vis absorbance spectra manifesting the iron redox cycling in the Fenton reaction. Group I: Fe^2+^, II: Fe^3+^, III: Fe^3+^ + EGCG, IV: Fe^2+^ + H_2_O_2_, V: Fe^3+^ + EGCG + H_2_O_2_, and VI: Fe^2+^ + *E. faecalis* culture medium. (f) SEM images of PS before (left) and after (right) EF@EGCG/Fe^3+^ co‐culture. Scale bar: 300 nm. (g) Size distributions of PS, *E. faecalis*, *E. faecalis* & PS co‐culture, EF@EGCG/Fe^3+^ and EF@EGCG/Fe^3+^ & PS co‐culture. (h) FT‐IR spectrum of PS (Group I), EF@EGCG/Fe^3+^ (Group II), and EF@EGCG/Fe^3+^ & PS co‐culture (Group III). (i) Schematic illustration of chemodynamic performance of EF@EGCG/Fe^3+^.

The hybrid functionality relied on the metabolic activity of *E. faecalis*. Growth curve analysis revealed an initial proliferation delay induced by the EGCG‑Fe^3+^ coating, followed by exponential growth after 12 h that surpassed the rate of unmodified E. faecalis and yielded a characteristic double‑S curve (Figure 2a). This is also supported by the colony formation assay, as it revealed a similar growth situation after 24 h of unmodified *E. faecalis* and EF@EGCG/Fe^3+^ (Figure 2b). Indeed, this peculiarity was conducive to the targeted colonization of EF@EGCG/Fe^3+^: during the transit through the digestive tract, the coated metal‐organic framework served as a protective shell, shielding *E. faecalis* from the corrosive effects of digestive fluids. A metal‐phenol network cellular coating comprised of polyphenols and Fe(III) has been proven to protect probiotics from the adverse effects of gastric acid [37].

The activation of EF@EGCG/Fe^3+^ is intrinsically coupled with the colonization and metabolic activity of *E. faecalis* in the intestinal environment. Upon successful colonization, *E. faecalis* continuously secretes H_2_O_2_ as a metabolic byproduct, and the gradual accumulation of localized H_2_O_2_ reaches the threshold required to initiate Fenton reactions, thereby driving the oxidative aging and agglomeration of NPs. The biogenic in situ generation of H_2_O_2_ plays a pivotal role in the functionality of EF@EGCG/Fe^3+^ hybrids. H_2_O_2_ production by *E. faecalis* was assessed using tetramethylbenzidine (TMB) colorimetry, in which horseradish peroxidase (HRP) catalyzed the reaction between TMB and H_2_O_2_ to yield blue ox‐TMB with an absorption peak at 652 nm. In the study, the presence of a distinct absorption peak at 652 nm and the observation of a blue solution confirmed the effective generation of H_2_O_2_ by *E. faecalis*, providing evidence for the occurrence of the Fenton reaction within the EF@EGCG/Fe^3+^ hybrids (Figure 2c). Following the generation of H_2_O_2_, it reacts with Fe^2+^, resulting from the reduction reaction of EGCG, and produces reactive oxygen species (ROS) with strong oxidizing properties, which is the foundational basis for inducing the aging of NPs. Thus, the production of ROS was also examined by TMB colorimetry. The colorimetric analysis presented a conspicuous absorption peak at 665 nm, which attenuated with the ascending concentration of EF@EGCG/Fe^3+^ hybrids (Figure 2d). An iron redox cycle involving multiple valence states was established through the coupled EGCG reduction and H_2_O_2_ oxidation reactions. This cycling was monitored by phenanthroline colorimetry. FeCl_3_ supplemented with EGCG produced an absorption peak and a reddish colour characteristic of Fe^2+^, whereas FeCl_2_ exposed to *E. faecalis* culture medium displayed a peak and a yellowish colour indicative of Fe^3+^. These observations verified the sustained Fenton catalytic performance of the EF@EGCG/Fe^3+^, with the ongoing iron cycling and continuously proliferating *E. faecalis* together ensuring a durable functional effect (Figure 2e and Figure S4).

To elucidate the impact of materials on NPs, 1 mg/mL polystyrene (PS) was co‐cultured with EF@EGCG/Fe^3+^ at 37°C for 3 days, and then PS was collected for SEM characterization. The untreated polystyrene (PS) exhibited a homogeneous distribution and a smooth, well‐defined surface. Conversely, upon co‐cultivation with EF@EGCG/Fe^3+^, the rough surface, blurred boundary, and agglomerating particles indicated the occurrence of aging and aggregation (Figure 2f). DLS measurements confirmed the increased particle size as well (Figure 2g). The alterations in functional groups in FT‐IR spectrum, particularly at ∼1640 cm^−1^ and ∼1400 cm^−1^, demonstrate the chemical aging process occurring on PS (Figure 2h and Figure S5). As shown in Figure 2i, upon oral administration, EF@EGCG/Fe^3+^ was disassembled in the intestine into *E. faecalis*, H_2_O_2_, and Fe^3+^. Reduction of Fe^3+^ to Fe^2+^ enabled a Fenton reaction with H_2_O_2_, generating ROS with strong oxidative capacity. The resulting ROS promoted the aging and agglomeration of PS, establishing the basis for subsequent NP excretion.

### 2.3 In Vivo Protective Effect and Biosafety of EF@EGCG/Fe^3+^

**FIGURE 3:**
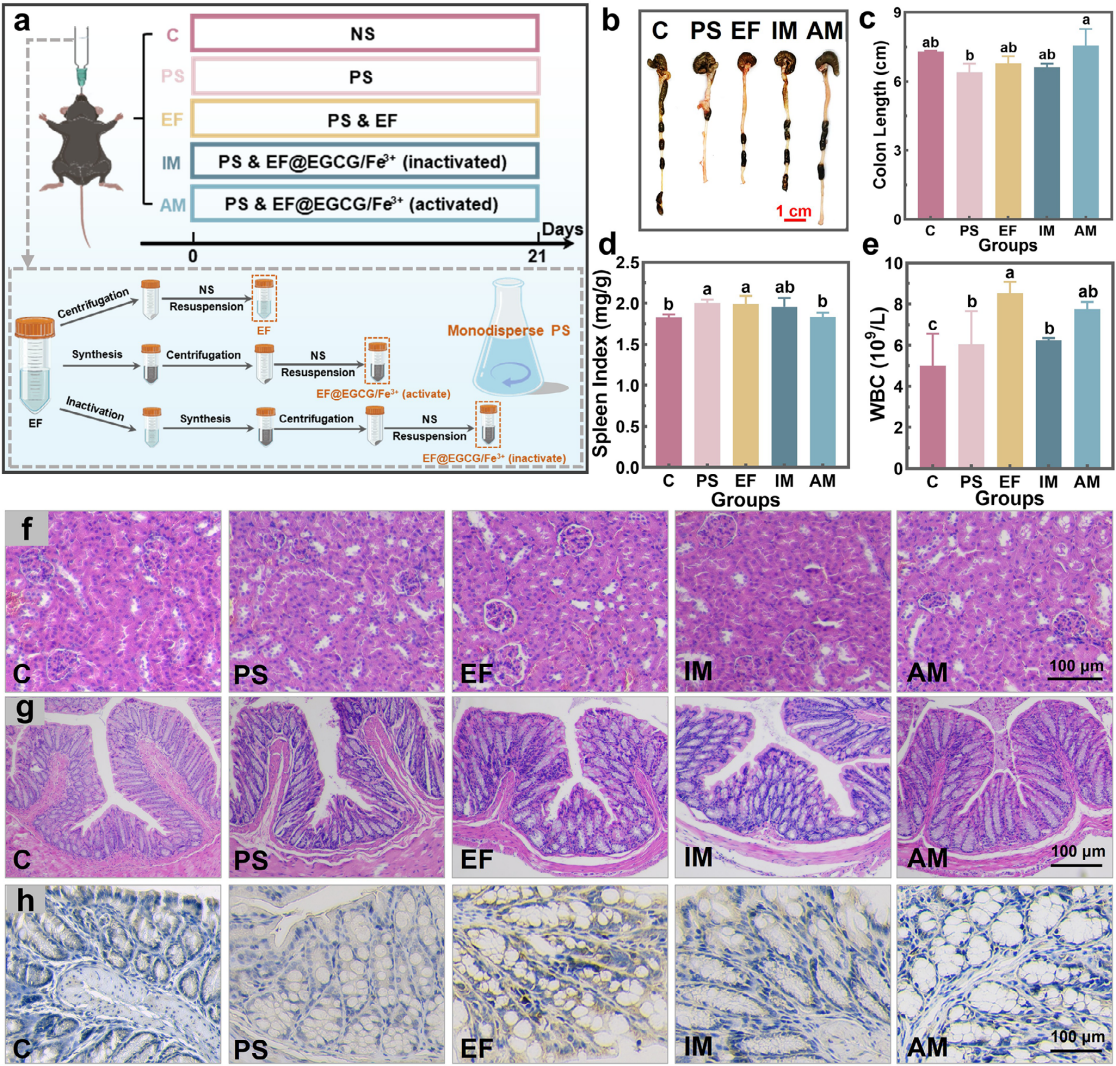
In‐vivo effect of EF@EGCG/Fe^3+^. (a) Schematic illustration of in vivo experiment. (b) Colon images, (c) length quantification, (d) spleen index, (e) WBC number, (f) H&E staining of kidney, (g) H&E staining of colon, and (h) immunohistochemical staining (IL‐6) of colon of various groups. Scale bar: 1 cm in (b) and 100 µm in (f), (g), and (h). For each group, *n* = 9 mice. Data represent the mean ± SD. Error bars with different letters indicate a significant difference according to one‐way ANOVA with Duncan and LSD test (*p* < 0.05).

Having confirmed the aggregation‐promoting effect of EF@EGCG/Fe^3+^ in vitro, the in vivo performance was evaluated. C57BL/6 mice exposed to PS received the EF@EGCG/Fe^3+^ biohybrid platform by intragastric administration, with the inactivated platform and unmodified *E. faecalis* used for comparison (Figure 3a). It is clearly indicated that EF@EGCG/Fe^3+^ best alleviated the intestinal atrophy induced by PS exposure, while showing no significant impact on the spleen index, lymphatic index, and lymph index (Figure 3b–d and Figure S6). Notably, these effects were accompanied by milder immune rejection, represented by WBC, in comparison to *E. faecalis* treatment, which is conducive to the colonization and subsequent therapeutic effects of the biohybrid platform (Figure 3e). Pathological analysis further explores the in vivo effect of EF@EGCG/Fe^3+^. From H&E staining of the kidney, compared with group PS, it was observed that the glomeruli in group AM exhibited uniform size, normal shape, and clear boundaries, as well as an obvious cystic cavity. This histological evidence substantiates the biocompatibility and biosafety of the hybrid material in comparison to group PS (Figure 3f). The same went for the other organs (Figure S7).

Moreover, from H&E slices of colons, EF@EGCG/Fe^3+^ treatment showed noticeable improvements in the mucosa and villi integrity, goblet cell vacuolation, and inflammatory cell infiltration induced by PS, surpassing the effects of *E. faecalis* and inactive biohybrid platform (Figure 3g). Additionally, the alleviation of intestinal inflammation by EF@EGCG/Fe^3+^ was further demonstrated through immunohistochemical staining, whereby the hybrid material effectively reversed the elevated expression of inflammatory factors induced by PS (Figure 3h and Figure S8). Taken together, the application of EF@EGCG/Fe^3+^ commendably mitigated intestinal injury caused by PS, as evidenced by the substantial restoration of colon morphology and the notable reduction in tissue inflammation.

To evaluate the in vivo biosafety of *E. faecalis* and EF@EGCG/Fe^3+^, mice were orally administered with the bacterium or the platform alone for 21 days. Results showed that body weight gain in each treatment group was comparable to that of the control group (Figure S9a). In Figure S9b, no significant differences were observed in serum liver/kidney function markers among groups (*p* > 0.05). H&E‐stained sections of heart, liver, spleen, lung, kidney, and colon revealed no obvious histopathological alterations (Figure S10). Collectively, these findings demonstrate the favorable biosafety of both *E. faecalis* and EF@EGCG/Fe^3+^ under the 21‐day exposure regimen.

### 2.4 EF@EGCG/Fe^3+^ Inhibited the Penetration of PS Into Intestine

**FIGURE 4:**
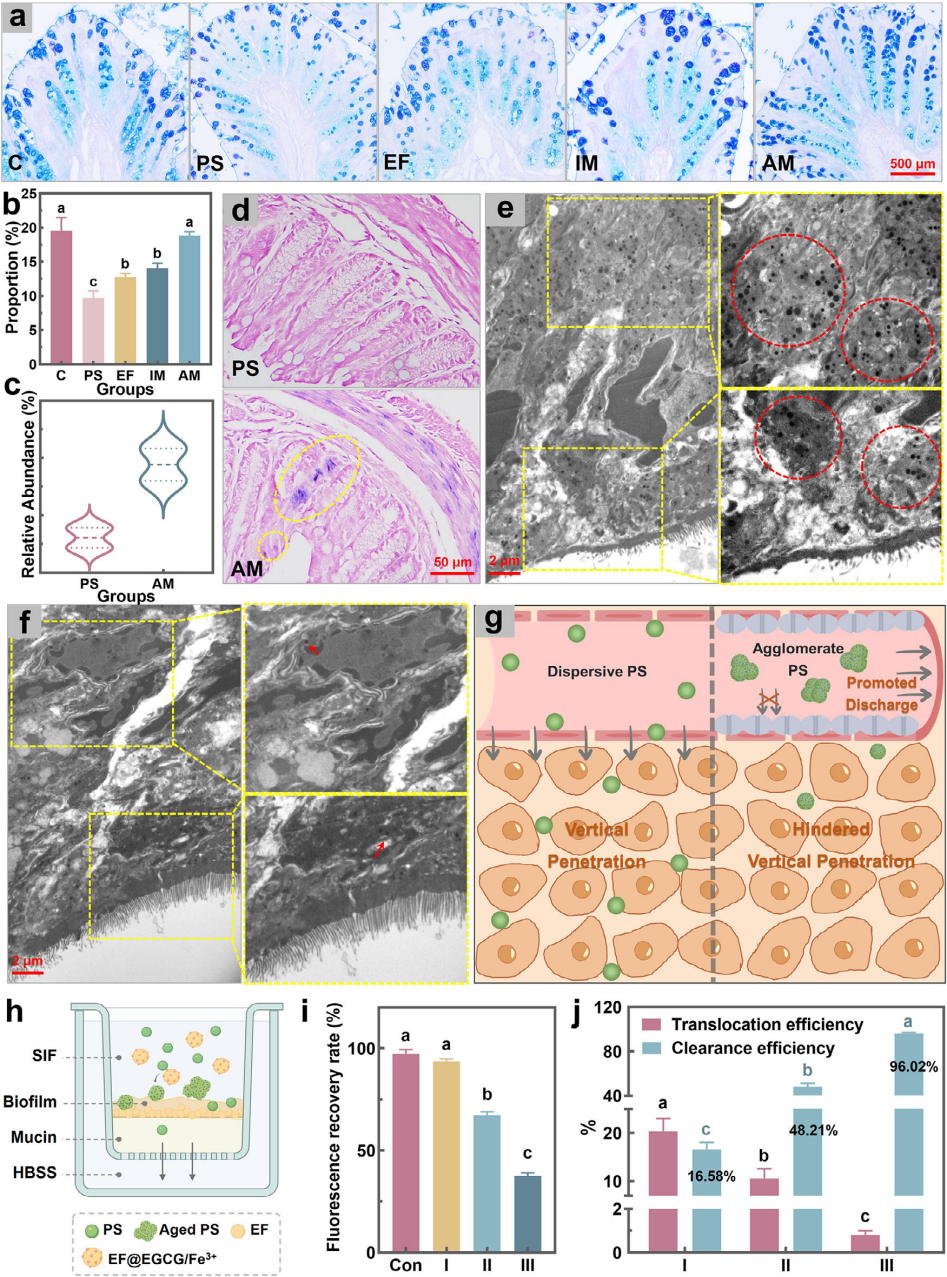
Antipenetration effect of EF@EGCG/Fe^3+^. (a) Images of AB‐PAS staining and (b) positive quantification of various groups. (c) Relative abundance of *E. faecalis* in group PS and AM. (d) Gram staining of colon in group PS and AM. TEM image of colon in group (e) PS and (f) AM. (g) Schematic illustration of intestinal penetration behavior of PS. For each group, *n* = 9 mice. (h) Schematic illustration of NPs penetration experiment. (i) The fluorescence recovery rate of PS. (j) The translocation efficiency and clearance efficiency of PS. Group Con: PS + PBS; I: PS + SIF; II: PS + SIF+ EF; III: PS + SIF+ EF@EGCG/Fe^3+^. Error bars with different letters indicate a significant difference according to one‐way or two‐way ANOVA with Duncan and LSD test (*p* < 0.05).

From AB‐PAS staining, goblet cells dyed blue have recovered by 93% in group AM, demonstrating that EF@EGCG/Fe^3+^ could effectively restore the impaired intestinal microstructure induced by PS (Figure 4a,b). Besides, Figure 4c revealed a significant increase in the abundance of *E. faecalis* after the administration of EF@EGCG/Fe^3+^ (Figure 4c), as well as represented via Gram staining (Figure 4d). These findings indicated that EF@EGCG/Fe^3+^ successfully colonized in the intestinal tract under adverse conditions, establishing the basis for its subsequent effects.

In vitro results revealed that PS exhibited aging and agglomeration when exposed to the oxidation of EF@EGCG/Fe^3+^. According to previous studies, larger particles would be blocked from penetrating the intestinal barriers on account of the size‐dependent effect [35, 36]. In vivo experiments substantiated the hypothesis. As observed from TEM, a large number of particles diffused and permeated into the muscular layer upon oral administration of PS, meanwhile, the integrity of the intestinal villus was seriously damaged (Figure 4e). Conversely, the administration of EF@EGCG/Fe^3+^ effectively ameliorated this situation. After EF@EGCG/Fe^3+^ treatment, the penetration of PS was substantially relieved, with only a few particles dispersed in the mucous layer and superficial muscular layer (Figure 4f). Remarkably, the structure of the intestinal villi exhibited a more complete restoration. To confirm whether PS were agglomerated under the action of EF@EGCG/Fe^3+^ in vivo, the colonic contents were further examined by SEM (Figure S11). Although the NPs were enshrouded by digestive residues in group PS, individual spherical boundaries remained clearly distinguishable. In contrast, PS in group AM were extensively fused with the surrounding colonic matrix into dense, inseparable clusters, in which sporadic spherical contours could be identified. These observations provide direct morphological evidence that EF@EGCG/Fe^3+^ actively drives the agglomeration of dispersed NPs within the intestinal tract. Immunofluorescence staining of colonic tissues showed that PS exposure markedly reduced Occludin‑1 expression compared with the group C, whereas the group AM restored Occludin‑1 expression to a level approaching that of the Control (Figure S12). Overall, as illustrated in Figure 4g, treatment with EF@EGCG/Fe^3+^ distinctly facilitated the agglomeration of dispersed PS, then presented a significant obstacle to the permeation. Simultaneously, the colonized *E. faecalis* acted as a bio‐barrier against PS, ultimately alleviating its detrimental effects on the intestine.

To evaluate the clearance efficiency of EF@EGCG/Fe^3+^ and the interception ability of the reinforced biofilm against PS, an in vitro experiment of PS translocation across the mucus layer was designed in Transwell (Figure 4h). The fluorescence recovery rate after 24 h of co‐incubation to assess the stability of the PS within the complex microbial environment. While the Control and Group I maintained high recovery rates (> 93%), the recovery rate dropped to 67.19% in Group II and plummeted to 37.57% in Group III (Figure 4i). This loss of signal in Group II is likely due to the metabolic activity of proliferating *E. faecalis*, while the severe quenching in Group III is attributed to Fenton reactions that degrade or quench the PS fluorescence, a phenomenon consistent with previous reports on metal‐polyphenol systems [38]. The PS translocation decreased to 10.59% after EF treatment, yielding a physical clearance efficiency of 48.21% (Figure 4j). Crucially, Group III exhibited a low translocation efficiency of only 0.80%. The results elucidate the dual mechanism of the EF@EGCG/Fe^3+^ in preventing NPs translocation: The EF colonization fortifies the intestinal barrier to physically hinder NP passage. Meanwhile, the Fenton reaction triggered by EF@EGCG/Fe^3+^ promotes PS aging, leading to an intrinsically decrease in PS permeability. The combination of these effects resulted in the interception of approximately 96.02% of NPs.

### 2.5 EF@EGCG/Fe^3+^ Facilitated the Elimination of PS

**FIGURE 5:**
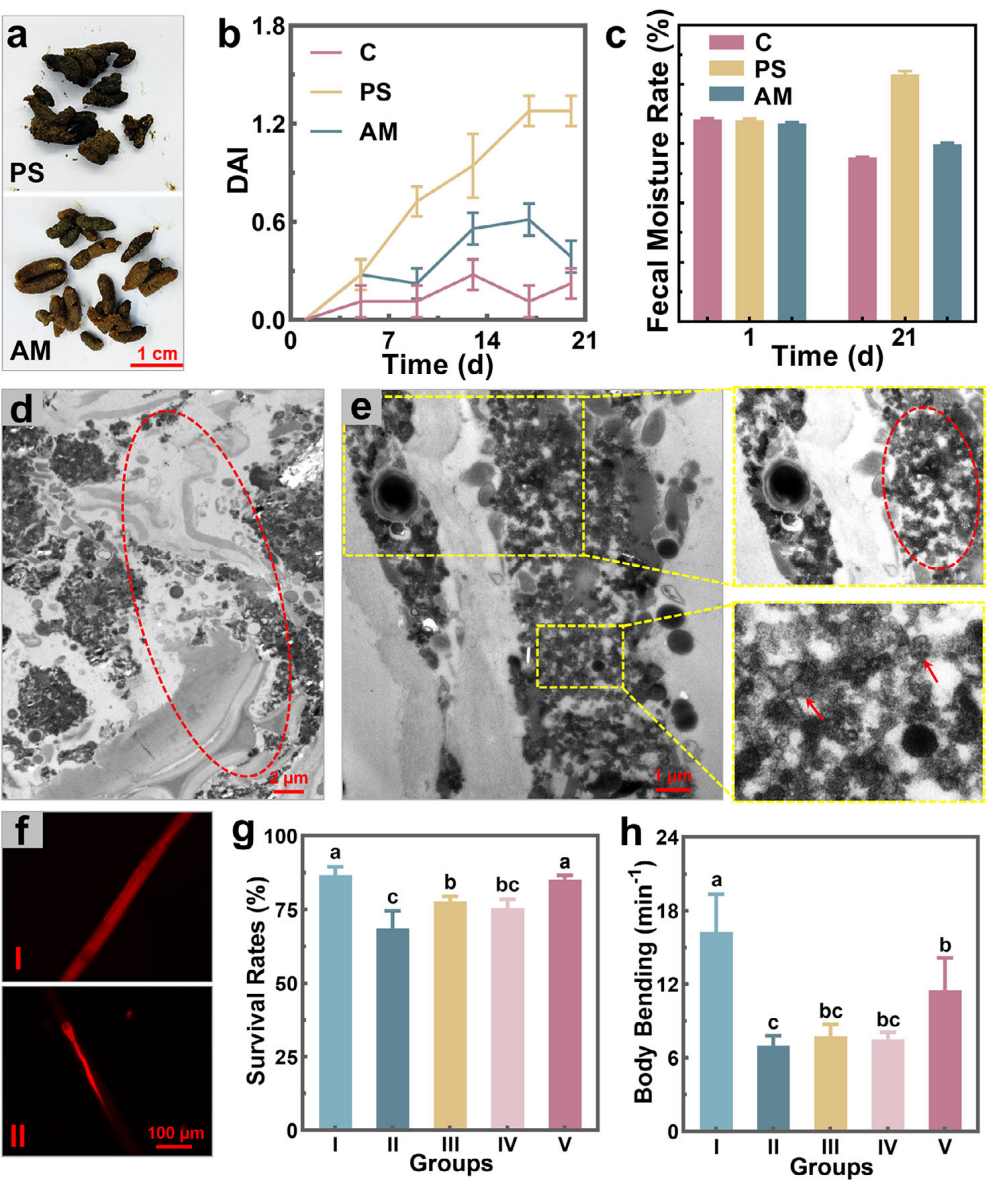
Excretion promotion effect of EF@EGCG/Fe^3+^. (a) Fecal images of group PS and AM. (b) DAI index and (c) fecal moisture rate of group C, PS, and AM. TEM images of fecal matter of group (d) PS and (e) AM. (f) Fluorescent images of I: PS and II: EGCG‐treated *C*. elegans. (g) Survival rates and (h) body bending frequency of I: C, II: PS, III: EF, IV: IM, V: AM. For each group, *n* = 9 mice in (b) and (c), and *n* = 5 *C. elegans* in (g) and (h). Error bars with different letters indicate a significant difference according to one‐way ANOVA with Duncan and LSD test (*p* < 0.05).

Under the action of EF@EGCG/Fe^3+^, the agglomerated PS deposits were ultimately expelled from the gastrointestinal tract with food residues. The collected feces were analyzed accordingly. The feces of mice in group PS exhibited distorted shape and high moisture content, distinct from those in group AM with normal shape and texture (Figure 5a,b). The DAI scores also reflected the gradual deterioration of intestinal health in group PS compared with group AM (Figure 5c). To gain further insights into the morphology of PS in excrement samples, feces were promptly collected and subjected to TEM. Consistent with the visual appearance, feces in group PS were characterized by a watery and loose texture, with limited presence of particles (Figure 5d). In stark contrast, the fecal matter from group AM exhibited a compact and hard texture, with a plethora of nano‐scale particles aggregating in clusters. High‑magnification imaging revealed a rough and jagged surface on these particles, confirming the occurrence of aging as demonstrated in vitro (Figure 5e).

Due to the transparent body and simple intestinal structure, *Caenorhabditis elegans* (*C. elegans*) serves as an ideal model organism suitable for investigating the migration and transformation behavior of PS within the intestinal tract. Fluorescent PS were introduced to *C. elegans* and subsequently monitored by fluorescence microscopy. With the increase of PS concentration, a gradual decline in the survival rate of *C. elegans* was observed (Figure S13). Following a 24‐h co‐culture with 2 µg/mL PS, the entire *C. elegans* emitted red fluorescence upon exposure to the excitation wavelength, which indicated that PS had effectively permeated the intestinal tract and disseminated homogeneously throughout the organism. While in the worms pretreated with EGCG, the total fluorescence intensity exhibited a significant decrease and was primarily confined to the intestinal tract (Figure 5f). A similar phenomenon appeared after activated EF@EGCG/Fe^3+^ treatment. The fluorescence intensity of group AM was slightly higher than that of group C, but there was no significant difference, and significantly lower than that of group PS (Figures S14 and S15). As the faded fluorescence illustrates the apparent removal of PS, along with the ameliorative survival rates, body bending and head swinging frequency (Figure 5g,h and Figure S16). Therefore, the experiment in *C. elegans* once again confirmed the superior effect of EF@EGCG/Fe^3+^ against PS.

### 2.6 EF@EGCG/Fe^3+^ Facilitated the Elimination of Polypropylene NPs

**FIGURE 6:**
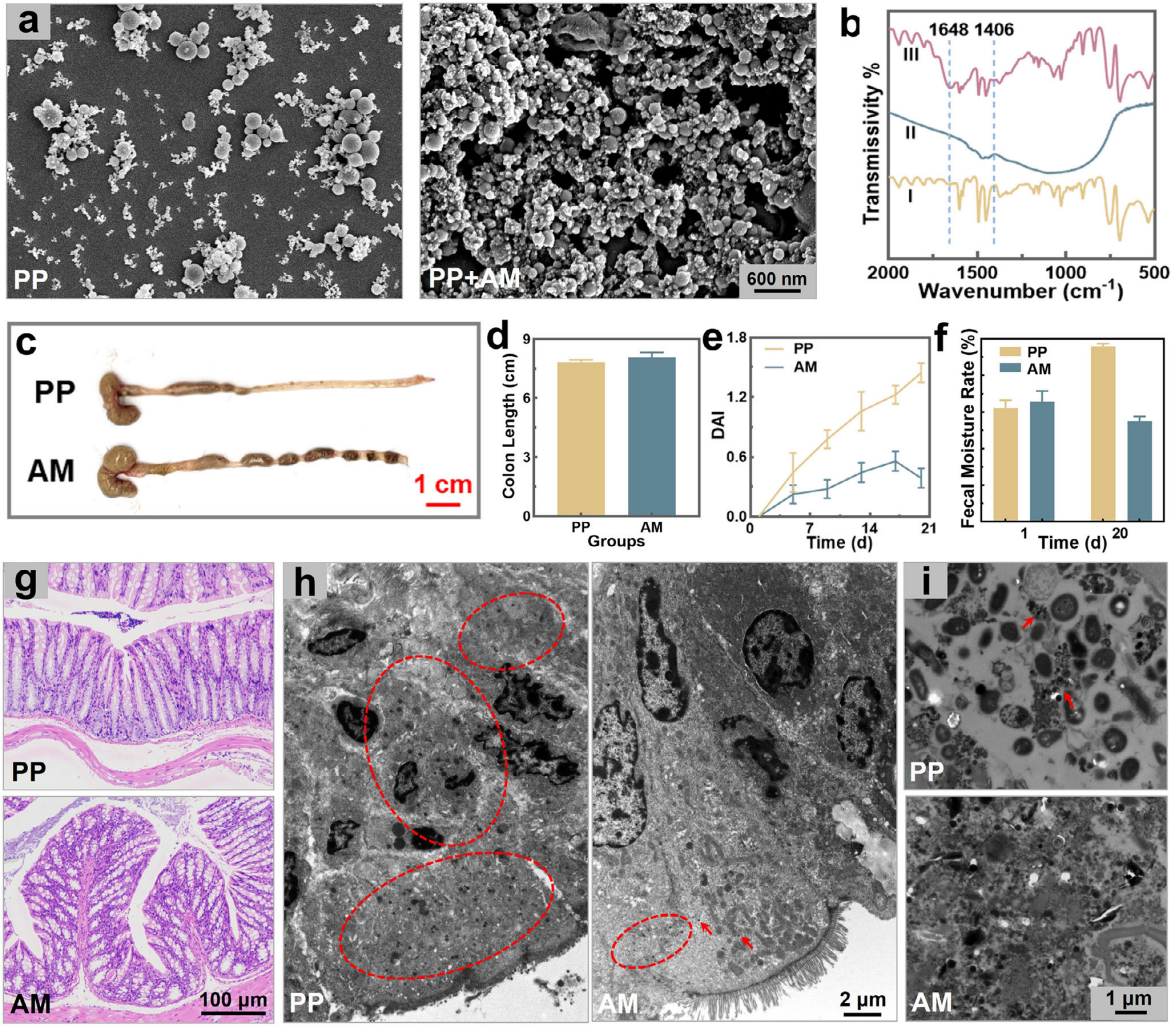
In vitro and vivo of EF@EGCG/Fe^3+^ against PP treatment. (a) SEM images of PP before (left) and after (right) EF@EGCG/Fe^3+^ co‐culture. (b) FT‐IR spectrum of PP, EF@EGCG/Fe^3+^, and EF@EGCG/Fe^3+^ & PP co‐culture. (c) Colon images, (d) length quantification, (e) DAI index, (f) fecal moisture rate of group PP and AM. (g) H&E staining of the colon of group PP and AM. TEM images of (h) colon and (i) feces of group PP and AM.

In virtue of exoteric, *C. elegans* and mice model, the effect of EF@EGCG/Fe^3+^ in combating PS has been certified. However, further investigation is required to ascertain whether EF@EGCG/Fe^3+^ demonstrates similar effects on other types of NPs and explore the acting pathway. Therefore, we adopted another kind of NPs—polypropylene (PP)—as experimental materials, which also constitutes a significant portion of environmental NPs [39]. Both in vitro and in vivo experiments demonstrated that EF@EGCG/Fe^3+^ performed analogously on PP. Co‑incubation with EF@EGCG/Fe^3+^ induced agglomeration of initially dispersed PP, accompanied by surface roughening(Figure 6a). Analysis of FT‐IR spectrum indicated alterations in the chemical composition of treated PP, providing evidence for the occurrence of aging (Figure 6b). In vivo, administration of EF@EGCG/Fe^3+^ restored intestinal condition, as shown by increased colon length, improved DAI scores, and reduced fecal moisture content, and the impaired villus structure observed in colonic pathology was repaired (Figure 6c–g).

TEM examination of colon and fecal samples was performed to evaluate the influence of EF@EGCG/Fe^3+^ on PP intestinal behavior. Within expectation, in contrast to the clouds of nano‐particles diffusely distributing in the colon of group PP, there were sparser particles in group AM and mainly confined to the superficial layer (Figure 6h). A similar pattern was found in feces, where particles in the PP group were scarce and monodisperse, while the EF@EGCG/Fe^3+^ treatment produced visually conspicuous aggregates (Figure 6i). Overall, EF@EGCG/Fe^3+^ impeded internalization and promoted excretion of PP by accelerating aging and agglomeration, analogous to the mechanism observed for PS.

Therefore, our findings have uncovered the efficacy of EF@EGCG/Fe^3+^ in modulating the intestinal behavior of NPs. The action process consists of several steps: (1) the successful Fenton reaction triggered by EF@EGCG/Fe^3+^; (2) the aging of NPs under the influence of strong oxidants; (3) the agglomeration of aging NPs; and (4) the suppressive penetration and promoted elimination. On account of the fact that plastics, that is, high‐molecular polymer, possesses strong reducibility, which renders them susceptible to oxidation and resultant aggregation. Hence, EF@EGCG/Fe^3+^ has universal effect on NPs, which has been verified on two major NPs in the environment.

Ingested NPs encounter a complex biochemical environment within the gastrointestinal tract, where their colloidal behavior is shaped not only by digestive fluids but also by co‐ingested food matrices. Dietary proteins, lipids, and polysaccharides, together with digestive enzymes, adsorb onto NP surfaces to form a biomolecular corona that can markedly alter particle aggregation state, surface chemistry, and subsequent cellular interactions [40, 41]. This corona‐mediated modulation varies substantially with food matrix composition, particle size, and digestive phase, collectively contributing to the heterogeneous behavior of NPs in vivo [42, 43]. The aggregation that occurs under these conditions is driven primarily by DLVO type noncovalent interactions: low gastric pH screens electrostatic repulsion and allows van der Waals forces to dominate, while digestive enzymes and food proteins bridge particles via hydrogen bonding and electrostatic interactions [44, 45].

These non‑covalent associations are condition‑dependent and largely reversible, as demonstrated by a simulated digestion experiment. Briefly, PS suspensions (0.5 mg/mL) were mixed 1:1 with the homogenate of commercial maintenance feed for laboratory mice (GB 14924.3‐2010) and incubated for 1 h, with pristine PS serving as the control. Both mixtures were then sequentially incubated in simulated gastric fluid (SGF, 37 °C, 2 h, 100 rpm) followed by SIF (37 °C, 12 h, 100 rpm). Subsequent SEM and TEM imaging revealed that PS showed mild aggregation in simulated gastric fluid and largely redispersed upon transfer to simulated intestinal fluid, and that food matrix co‑incubation resulted in only a slight size increase to approximately 100 nm, attributable to protein corona formation, with particles remaining distinctly independent (Figure S17).

In contrast, hydroxyl radicals generated by EF@EGCG/Fe^3+^ chemically oxidize the PS backbone and introduce carbonyl and carboxyl groups, enabling inter‑particle hydrogen bonding and covalent crosslinking that yield dense, essentially irreversible aggregates [46, 47]. Previous studies have demonstrated that NPs aggregated during digestion, despite exhibiting reduced absorption efficiency, can still be internalized by intestinal epithelial cells and retain persistent cellular toxicity [43, 48]. This underscores that the weak and reversible agglomeration produced by gastrointestinal digestion is insufficient to prevent NP penetration and associated intestinal harm, highlighting the necessity of chemically aged aggregation driven by EF@EGCG/Fe^3+^, where covalent surface modification achieves robust and irreversible NP immobilization at the intestinal barrier.

However, a potential concern arises from the fact that the macroscopic intestinal environment normally maintains a near‐neutral to slightly alkaline pH (approximately 7–8), which appears incompatible with the acidic requirements of the classical Fenton reaction. This apparent contradiction is resolved by recognizing that *E. faecalis*, as a prolific lactic acid bacterium, continuously secretes organic acids during its growth and metabolic activity [49, 50]. The sustained release of these metabolites actively shapes a localized acidic microenvironment in the immediate vicinity of the colonized bacteria. This localized acidification aligns with the well‐established mechanism by which various probiotic strains antagonize enteric pathogens through the production of short‐chain fatty acids and the concomitant reduction of local luminal pH [51, 52]. Consequently, the lactic acid‐driven acidic microenvironment generated by *E. faecalis* supplies permissive local pH conditions that are sufficient for sustaining the EF@EGCG/Fe^3+^‐mediated Fenton reaction and the resulting NP aging and aggregation, without requiring acidification of the entire intestinal lumen.

Certain aspects of the present study warrant further consideration. The in vivo aggregation time and critical aggregation concentration of NPs remain difficult to quantify, given the large epithelial surface area of the intestinal tract in large mammals such as pigs, nonhuman primates, and humans, and the complex biochemical environment therein [53]. Yet this quantification challenge does not imply that the material would lose its ability to impede NP penetration at lower NP concentrations. The Fenton‐driven oxidative aging introduces carbonyl and carboxyl groups onto NP surfaces, enabling covalent and hydrogen‐bonding interactions with surrounding biomolecules, including digested food residues, mucins, and bacterial debris, to form heterogeneous aggregates [32]. This mechanism does not require direct NP‐NP contact to substantially enlarge the particle size and reduce permeability. In vitro data indicate that approximately 3 days (48 h biofilm pre‑culture plus 24 h clearance measurement) are required for *E. faecalis* colonization and EF@EGCG/Fe^3+^‑mediated interception to achieve near‑complete NP clearance (∼96%). The substantial disparity between the Transwell surface area and the human intestinal surface area further precludes direct quantitative extrapolation of barrier penetration rates. Given that probiotic colonization requires time to establish and that sustained H_2_O_2_ supply is needed, daily oral administration is recommended to maximize the preventive efficacy of the platform [54]. Future studies validating the efficacy of EF@EGCG/Fe^3+^ in large animal models are warranted to bridge this translational gap and to further evaluate its clinical feasibility.

## 3 Conclusions

This study develops a health risk prevention and control strategy designed to mitigate the in vivo hazards of NPs through regulating the transformation and migration of NPs within the intestine, rather than merely alleviating the downstream toxic effects. The edible biohybrid platform EF@EGCG/Fe^3+^ integrates colonized *E. faecalis* as an in situ H_2_O_2_ generator with an EGCG/Fe^3+^ complex that sustains iron redox cycling, driving continuous Fenton reactions to produce hydroxyl radicals. These radicals accelerate NP oxidative aging, increasing surface carbonyl and carboxyl groups that promote covalent and hydrogen‑bonding interactions with luminal biomolecules, resulting in the formation of micrometer‑scale heterogeneous aggregates. Concurrently, the intestinal barrier is reinforced through the gut‐repairing effects of *E. faecalis* and EGCG. The agglomerated NPs are sterically blocked from epithelial penetration and efficiently excreted with feces. The platform achieved a 96% NP clearance efficiency within 24 h and reduced the translocation rate to 0.80% in vitro. Daily oral co‑administration in mice significantly restored intestinal goblet cell abundance (∼93% of control), preserved tight junction protein Occludin‑1 expression, and facilitated NP agglomeration within the colonic lumen. The similar protective efficacy observed against two prevalent environmental NPs (PS and PP) prompts the potential universality of this strategy in mitigating NPs hazards. This study establishes a promising solution in nanotoxicology intervention, from postinjury remediation to the proactive regulation of pollutant behavior in vivo.

## 4 Experimental Section

### 4.1 Materials

Potassium chloride (KCl, 99.5%), sodium chloride (NaCl, 99.5%), and sodium hypochlorite (NaClO, ≥ 60.0%) were obtained from Aladdin Biochemical Technology Co., Ltd (Shanghai, China). Peptone, yeast extract, beef extract, glucose, diammonium citrate, tween‐80, magnesium sulfate (MgSO_4_, 99.0%), manganese sulfate (MnSO_4_, ≥ 99.0%), agar, and dipotassium phosphate (K_2_HPO_4_, ≥ 99.0%) were purchased from Yuanye Bio‐Technology Co., Ltd (Shanghai, China). Epigallocatechin gallate (EGCG, ≥ 98.0%) was purchased from Beyotime Biotechnology Co., Ltd (Shanghai, China). Ferric trichloride hexahydrate (FeCl_3_·6H_2_O, 99.0%), sodium acetate (NaAc, 99.0%), hydrogen peroxide (H_2_O_2_, 30.0%) and neutral formalin solution were gained from Macklin Biochemical Co., Ltd (Shanghai, China). 3, 3’, 5, 5’‐Tetramethylbenzidine (TMB) and horseradish peroxidase (HRP) were acquired from Sigma aldrich (Shanghai) trading Co., Ltd (Shanghai, China). Phosphate buffer saline (PBS) and normal saline (NS) were obtained from Dalian Meilun Biotechnology Co., Ltd (Liaoning, China). Nonfluorescent (25 mg/mL) and red fluorescent (10 mg/mL) PS‐NP beads, each with a diameter of 80 nm, were sourced from Tianjin Baseline ChromTech Research Centre (Tianjin, China). Other chemical reagents were obtained from Sinopharm Chemical Reagent Co., Ltd (Shanghai, China).

### 4.2 Characterization

The morphology of materials and distribution of elements were observed with a thermal field emission scanning electron microscope (TFESEM, ZEISS GeminiSEM 300) and a transmission electron microscope (TEM, Hitachi H‐7650). Fourier infrared transform spectroscopy (FTIR) spectra were obtained using an infrared spectrometer (NICOLET iS50FT‐IR, Thermo Scientific). Particle size distributions and zeta potentials of materials were detected by a zetasizer (Nano‐ZS90, Malvern). Ultraviolet visible (UV–vis) spectra were acquired by a Multiscan Spectrum (Infinite 200 Pro, Tecan).

### 4.3 Culture of *E. faecalis*

Sterile liquid MRS medium was prepared to culture *E. faecalis* (with 10 g peptone, 5 g yeast extract, 10 g beef extract, 20 g glucose, 5 g NaAc, 2 g diammonium citrate, 1 mL tween‐80, 0.58 g MgSO_4_, 0.05 g MnSO_4_ and 2 g K_2_HPO_4_ dissolved in 1 L deionized water), and 15 g agar was extra added to obtained solid MRS medium. *E. faecalis* employed in this work is a commercially available probiotic strain, purchased from a food ingredient processing plant, with the number CGMCC 1.15424. After being purified from the solid medium, *E. faecalis* was rotary shaken in liquid medium in a homothermic shaking incubator (37°C, 200 rpm) for 24 h before formal experiments.

### 4.4 Synthesis of EF@EGCG/Fe^3+^

EF@EGCG/Fe^3+^ was hierarchically synthesized via electrostatic adsorption and self‐deposition. 1 mL EGCG solution (2 mg/mL) was dropwise added to 10^7^ CFU *E. faecalis* dispersed in 8 mL deionized water and stirred for 0.5 h at room temperature (RT), then 1 mL FeCl_3_ solution (2 mg/mL) was also dripped into the mixed solution and stirred for 0.5 h at RT in the same way. After repeated lavation, synthesized EF@EGCG/Fe^3+^ was finally gathered by centrifugation and then re‐suspended in ultrapure water for future usage.

### 4.5 Redox Cycling of Iron Element

Phenanthroline combined with Fe^2+^ could produce an orange complex. In order to directly represent the iron cycling in the working process of EF@EGCG/Fe^3+^, phenanthroline colorimetry was applied, and the following groups were set up: I. Fe^2+^; II. Fe^3+^; III. Fe^3+^ + EGCG; IV. Fe^2+^ + H_2_O_2_; V. Fe^3+^ + EGCG + H_2_O_2_; VI. Fe^2+^ + *E. faecalis* culture medium. Their UV–vis absorption was measured between 300 and 600 nm.

### 4.6 ROS Identification

To represent the generation of H_2_O_2_ and ROS, TMB reagent was applied. In a strong oxidizing environment, colorless TMB could be oxidized into blue oxidized TMB (Ox‐TMB), so the TMB chromogenic method was adopted to detect the production of H_2_O_2_ and ·OH in this study. To investigate the H_2_O_2_‐producing capacity of *E. faecalis*, 300 µL HRP water solution (1 U/mL) and 500 µL culture supernate of *E. faecalis* were successively added into 1.5 mL PBS solution (pH 5.8). After reacting for 10 min, 300 µL TMB ethanol solution (8 mM) was appended, and the UV–vis absorption between 325 and 825 nm was measured. Analogically, ·OH can oxidize colorless TMB into oxidized TMB (Ox‐TMB) that presents an absorption peak near 652 nm, so a similar method was used to detect ·OH produced by EF@EGCG/Fe^3+^. Different concentrations of EF@EGCG/Fe^3+^ suspension (0, 100 µg/mL, and 200 µg/mL) were incubated with TMB (1.6 mM), subsequently, the UV–vis spectra at 652 nm were recorded.

### 4.7 Mice Feeding and Grouping

Male C57BL/6 mice (6–8 weeks old, 20 ± 2 g) were purchased from Hangzhou Sailojin Biotechnology Co., Ltd (Hangzhou, China). The animal experiment was performed complying with the Guidelines for Care and Use of Laboratory Animals of Zhejiang University and approved by the Experimental Animal Welfare Ethics Committee of Zhejiang University (ZJU20230513). In formal experiment, based on the completely randomized design, mice were randomly assigned into five groups (*n* = 9): (1) C (NS); (2) PS (5 mg/kg PS suspension); (3) EF (5 mg/kg PS suspension with 10^11^ CFU/kg *E. faecalis*); (4) IM (5 mg/kg PS suspension with 10^11^ CFU/kg inactivated EF@EGCG/Fe^3+^); (5) AM (5 mg/kg PS suspension with 10^11^ CFU/kg activated EF@EGCG/Fe^3+^). The inactivated EF@EGCG/Fe^3+^ was prepared as follows: *E. faecalis* suspension at the logarithmic growth phase was autoclaved at 121°C for 20 min, and then the inactivated bacteria were used in place of live bacteria in the same synthetic procedure as for EF@EGCG/Fe^3+^ to obtain the inactivated EF@EGCG/Fe^3+^.

All groups were conducted intragastric administration once a day at a fixed time for 21 days. Fecal conditions were monitored daily, including disease activity index (DAI) assessment and moisture content determination. On the last day, before sacrifice, blood from the mice eye sockets was gathered and conducted blood routine analysis, including white blood cell count (WBC) and lymphocyte index (Lymph). Then euthanasia was performed, and colons, lymph and spleens were immediately collected and preserved for further analysis.

### 4.8 Histological Examination

After 21 days of treatment, colon tissues were collected and fixed in 10% neutral formalin solution. Through dehydrated, embedded, and sliced, the samples were performed H&E, Alcian blue‐periodic acid Schiff (AB‐PAS), and Gram staining, as well as subjected to TNF‐α, IL‐1β and IL‐6 labeling. The stained slices were also observed under Ts2‐FL.

### 4.9 Biosafety Assessment

To evaluate the biocompatibility of materials, mice were randomly divided into three groups (*n* = 3) and orally administered with equal volumes of distilled water (Group Con), *E. faecalis* suspension (1 × 10^11^ CFU/kg), or EF@EGCG/Fe^3+^ suspension (1 × 10^11^ CFU/kg) once daily for 21 consecutive days. Body weight was recorded during the treatment period. After the final administration, mice were euthanized, and blood was collected for serum liver/kidney function markers (ALT, AST, ALP, BUN). Heart, liver, spleen, lung, kidney, and colon tissues were harvested for histopathological examination following H&E staining.

### 4.10 Intestinal Flora Relative Abundance of *E. faecalis*

After taking fresh intestinal content samples, extraction, amplification, purification, and sequencing were performed, and the data were analyzed on the online platform of Majorbio Biomedicine Technology Co., Ltd (Shanghai, China).

### 4.11 Evaluation of the NP Clearance Efficiency of EF@EGCG/Fe^3+^

An Transwell model was established by precoating the upper chambers with a 1.0% (w/v) mucin solution. *E. faecalis* was inoculated and incubated for 48 h to preform a microbial biofilm on the mucin surface, after which excess medium was removed, and HBSS was added to the lower chambers. The total liquid volume in the upper chamber for all groups was standardized to 500 µL. Fluorescent PS (Ex/Em: 620/680 nm, 0.25 mg/mL) was introduced to all upper chambers. The specific treatments in the upper chambers, built upon the preformed biofilm and mucin layer, were as follows: Group Con: PS + PBS; I: PS + simulated intestinal fluid (SIF, pH 7.0); Group II: PS + SIF + EF suspension (10^8^ CFU/mL); Group III: PS + SIF + EF@EGCG/Fe^3+^ suspension (containing 10^8^ CFU/mL EF). The initial fluorescence intensity of the PS NPs administered to the apical chamber at 0 h was measured and designated as F_0_. In consideration of potential fluorescence quenching induced by the materials, parallel reference groups (treated identically to Group II and III, respectively) were simultaneously established in 12‐well plates without Transwell inserts to serve as the fluorescence baselines (F_1_). After 24 h of co‐incubation, liquids from both chambers of the Transwell and the 12‐well plates were collected (F_upper_ and F_lower_). The barrier performance and NP degradation were quantitatively evaluated using the following equations:

$$Recovery\;Rate\;\left(\%\right)=\left[\;\left(F_{upper}+F_{lower}\right)\;/\;F_{0}\right]\;\times \;100\%$$

$$Translocation\;Efficiency\;\left(\%\right)=\left(F_{\mathrm{lower}}\;/\;F_{1}\right)\;\times \;100\%$$

$$\begin{array}{ccc} & & \mathrm{Clearance}\;\mathrm{Efficiency}\;\left(\%\right)\\ & & \;=\left[\left(\mathrm{Translocation}\;\mathrm{Efficienc}y_{\mathrm{Group}\;\mathrm{Con}}\right.\right.\\ & & \;-\left.\left.\mathrm{Translocation}\;\mathrm{Efficienc}y_{\;\mathrm{Treatment}}\right)\right.\\ & & \;\left./\;\mathrm{Translocation}\;\mathrm{Efficienc}y_{\mathrm{Group}\;\mathrm{Con}}\right]\;\times \;100\% \end{array}$$

For group Con and group I without quenching, F_0_ was used as F_1_.

### 4.12 *C. elegans* Culture and Treatments

N2 strains were cultured on standard nematode growth medium (NGM, 2.5 g peptone, 3 g NaCl, and 20 g agar) or K medium (32 mM KCl, 51 mM NaCl). After synchronization, *C. elegans* were raised from L1 phase to adult on NGM medium with *Escherichia coli* OP50 (OP50), *E. faecalis* (EF), inactivated EF@EGCG/Fe^3+^ (IM), and activated EF@EGCG/Fe^3+^ (AM) as food. Then, adult worms were cultured in K medium containing 1 µg/mL PS for 24 h. Worms fed only OP50 served as the control group (C). After exposure, the survival rate and locomotion behaviors were evaluated. To observe the in vivo accumulation of PS, the nematodes were fed fluorescent PS and observed with a fluorescence microscope.

### 4.13 Statistical Analysis

All data were obtained through repeated experiments (*n* ≥ 3) and expressed as means ± SD. Statistical analysis was conducted by SPSS 19.0 of One‐Way ANOVA followed by the t‐test to assess statistical differences among different groups. The difference between any two groups represented by different letters in the histogram was statistically significant (*p* < 0.05). All fluorescence images were quantified by Image Pro Plus 6.0 software.

## Author Contributions

**Su Zhou**: writing the original draft, conceptualization, formal analysis, methodology, data curation, visualization, resources. **Anran Yan**: writing, review and editing, writing the original draft, formal analysis, validation, data curation. **Haowei Guo**: validation, formal analysis, supervision. **Ping Chen**: project administration, writing, review and editing, supervision. **Qiang Chu**: conceptualization, project administration, resources, writing, review and editing, supervision, funding acquisition.

## Conflicts of Interest

The authors declare no conflicts of interest.

## Supporting information




**Supporting File**: advs75918‐sup‐0001‐SuppMat.docx.

## Data Availability

The data that support the findings of this study are available from the corresponding author upon request.
