# Supplementary material for: An Edible Biohybrid Platform Accomplishes In Situ Fenton‐Mediated Enteral Nanoplastics Aging and Excretion
Source: Adv Sci (Weinh). 2026 May 30;13(48):e75918. doi: 10.1002/advs.75918 (PMC13337115; doi:10.1002/advs.75918)
Supplement: Supplementary file 1 [file ADVS-13-e75918-s001.docx]

**Supporting Information**

**An edible biohybrid platform accomplishes in situ Fenton-mediated enteral nanoplastics aging and excretion**

*Su Zhou, Anran Yan, Haowei Guo, Ping Chen , Qiang Chu**

Su Zhou, Anran Yan, Haowei Guo, Ping Chen, Qiang Chu

Tea Research Institute, Zhejiang University, Hangzhou 310058, China

*Corresponding author: Qiang Chu. Email: [0619363@zju.edu.cn](mailto:0619363@zju.edu.cn)

Su Zhou and Anran Yan contributed equally to this work.

**Figure S1.** SEM image of *E. faecalis*.

**Figure S2.** (a) TEM image of the minerals on *E. faecalis*. (b) XRD spectrum of *E. faecalis*, EF@EGCG and EF@EGCG/Fe^3+^.

**Figure S3.** (a) Image of *E. faecalis* and EF@EGCG/Fe^3+^ suspension. (b) UV-vis spectrum of *E. faecalis*, EGCG, EF@EGCG and EF@EGCG/Fe^3+^. (c) FT-IR spectrum of *E. faecalis*, EGCG and EF@EGCG/Fe^3+^.

**Figure S4.** Image of phenanthroline colorimetry in group I: Fe^2+^, II: Fe^3+^, III: Fe^3+^ + EGCG, IV: Fe^2+^ + H_2_O_2_, V: Fe^3+^ + EGCG + H_2_O_2_, and VI: Fe^2+^ + *E. faecalis* culture medium.

**Figure S5.** FT-IR spectrum of PS, EF@EGCG/Fe^3+^ and EF@EGCG/Fe^3+^ & PS co-culture.

**Figure S6.** (a) Lymphatic index and (b) lymph index of group C, PS, EF, IM and AM. Error bars with different letters indicate a significant difference according to one-way ANOVA with Duncan and LSD test (*p* < 0.05).

**Figure S7.** H&E staining of heart, liver, spleen and lung of group C, PS, EF, IM and AM.

**Figure S8.** Immunohistochemical staining (IL-1β and TNF-α) of colon of various groups.

**Figure S9.** Body weight (a) and serum liver/kidney function markers (b) of group Con, *E. faecalis* or EF@EGCG/Fe^3+^. Each treatment group contained 3 mice.

**Figure S10.** H&E staining of colon, liver, heart, spleen, lung and kidney of group Con, E. faecalis and EF@EGCG/Fe^3+^.

**Figure S11.** SEM images of PS in the colon contents of mice. Group C: Pure colon contents; group PS: Fed with PS; group AM: Fed with PS and EF@EGCG/Fe^3+^.

**Figure S12.** Immunofluorescence staining of Occludin‑1 (red) in colonic tissue sections. Nuclei were counterstained with DAPI (blue). Group C: pure water; PS: 5 mg/kg PS suspension; AM: 5 mg/kg PS suspension with 10^11^ CFU/kg activated EF@EGCG/Fe3+.

**Figure S13.** (a) Survival rates after treated with 0, 0.5, 1, 2, 4, 6 μg/mL PS. (b) Optical images of I: PS and II: AM treated *C.* elegans. Error bars with different letters indicate a significant difference according to one-way ANOVA with Duncan and LSD test (*p* < 0.05).

**Figure S14.** Optical images and corresponding fluorescence images of in group I: C, II: PS, III: EF, IV: IM and V: AM.

**Figure S15.** Analysis of relative fluorescence intensity in in group I: C, II: PS, III: EF, IV: IM and V: AM. The fluorescence intensity is normalized with group I as the control. Error bars with different letters indicate a significant difference according to one-way ANOVA with Duncan and LSD test (*p* < 0.05).

**Figure S16.** Head swinging frequency of I: C, II: PS, III: EF, IV: IM, V: AM. Error bars with different letters indicate a significant difference according to one-way ANOVA with Duncan and LSD test (*p* < 0.05).

**Figure S17.** SEM and TEM images of pristine PS (a) and the mixture of PS and food matrix (b). Both groups were subjected to of SGF (2h) and SIF (12 h) digestion. The food matrix is composed of the homogenate of commercial maintenance feed for laboratory mice.
